# Supplementary material for: Are Leading Risk Factors for Cancer and Mental Disorders Multimorbidity Shared by These Two Individual Conditions in Community-Dwelling Middle-Aged Adults?
Source: Cancers (Basel). 2020 Jun 26;12(6):1700. doi: 10.3390/cancers12061700 (PMC7352661; doi:10.3390/cancers12061700)
Supplement: Supplementary file 1 [file cancers-12-01700-s001.pdf]

cancers

MDPI

Article

Are Leading Risk Factors for Cancer and Mental Disorders Multimorbidity Shared by These Two Individual Conditions in Community-Dwelling Middle-Aged Adults?

Xianwen Shang, Allison M. Hodge, Wei Peng, Mingguang He, and Lei Zhang

Supplementary Materials

Text S1. Classification of Independent Variables

This section describes how the baseline data for independent variables were collected and the classification of these variables.

Participants were classified as current, former and never smokers based on two questions: "Have you ever been a regular smoker?" and "Are you a regular smoker now?". Passive smoking was self-reported in hours per week at home or in other places and was divided in two groups as passive smoking or not.

Alcohol intake was calculated based on two questions: "About how many alcoholic drinks do you have each week?" and "On how many days each week do you usually drink alcohol?". Responses were then categorized according to number of drinks per week (0, 1–4, 5–7, 8–14, or >14) using the National Health and Medical Research Council definitions, with more than two drinks per day (14 drinks/week) considered as consuming "risky" levels of alcohol.

Physical activity was measured using the Active Australia Survey where the total time one spent on walking, moderate-intensity, and vigorous-intensity physical activity (bouts of at least 10 min) in the previous week was assessed [1]. Questions were also asked about number of hours spent on sitting, watching television, and sleep in a typical 24-h day.

Sleep and sitting time were assessed using the following question: "About how many hours in each 24 h day do you usually spend doing the following: sleeping and sitting?". Sleep duration was divided into three groups: <7, 7–9, and >9 h. Sitting time was categorized into two groups as ≤8 or >8 h per day.

Outdoor time per day was assessed using the following question: "About how many hours a day would you usually spend outdoors on a weekday and on the weekend?". An average outdoor time per day in one week in hours was calculated based on the time spent on workdays and weekend days, and it was categorized into five groups according to the quintiles.

Frequency of dietary intakes including vegetable, fruit, breakfast cereal, milk, fish, chicken, red meat, and processed meat per week or per day was recorded based on separate questions. Vegetable intake was divided into five groups according to the quintiles based on the following question: "About how many serves of vegetables do you usually eat each day? A serve is half a cup of cooked vegetables or one cup of salad". Fruit intake was divided into four groups: none, one serving/day, two servings/day, and three or more servings/day based on the following question: "About how many serves of fruit or glasses of fruit juice do you usually have each day? A serve is one medium piece or two small pieces or one cup of diced or canned fruit pieces". Fish intake was divided into three groups: none, one serving per week, and two or more servings per

week based on the following question: "About how many times each week do you eat?". The same question was asked for chicken, red meat, and processed meat. Milk intake was categorized as none, skimmed fat/reduced fat/soy milk, and whole milk based on the following question: "Which type of milk do you mostly have?". Breakfast cereal was defined as non-high-fiber and high-fiber groups based on the following question: "If you eat breakfast cereal, is it usually bran cereal (all bran, bran flakes, etc.), muesli, biscuit cereal (weetbix, shredded wheat etc.), oat cereal (porridge, etc.), or other (cornflakes, rice bubbles, etc.)?".

Socioeconomic status was also assessed using the Index of Relative Socio-economic Disadvantage according to postcode that ranks the income, qualifications, and skilled occupations of residents within an area [2]. Participants were divided into five groups according to the quintiles of Index of Relative Socio-economic Disadvantage, with the lowest quintile representing the greatest socio-economic disadvantage. Health insurance was divided into four groups: private with extras, private no extras, healthcare concession, and none of the above.

Geographic remoteness was divided into four groups including major cities, inner regional area, outer regional area, and remoteness using the Accessibility Remoteness Index of Australia [3].

Psychological distress was accessed using the Kessler-10 scale [4], which provides a global measure of anxiety and depressive symptoms experienced in the preceding month. Scores range from 10 to 50, with the following categories: low (10–11), mild (12–15), moderate (16–21), and high (22–50) psychological distress.

Self-reported quality of life was classified as excellent, very good, good, fair, or poor based on the following question: "In general, how would you rate your quality of life?".

Self-reported overall health was classified as excellent, very good, good, fair, or poor based on the following question: "In general, how would you rate your overall health?".

Four questions from the Duke Social Support Scale asked the respondent how many times per week they spend time with friends or family they do not live with (0, 1–2, and ≥3 were scored as 1, 2, and 3, respectively), talk to someone (friends, relatives or others) on the telephone (0–1, 2–5, and ≥6 were scored as 1, 2, and 3, respectively), spend time at meetings of social clubs, religious/other groups (0–1, 2–5, and ≥6 were scored as 1, 2, and 3, respectively), and how many people outside home, within 1 h of travel they can depend on or feel very close to (0, 1–2 and ≥3 were scored as 1, 2, and 3 respectively) [5]. The total social interaction score ranged from 4–12 and was categorized as low (4–6), moderate (7–9), and high levels (10–12).

Family history of chronic diseases including heart disease, stroke, hypertension, cancer, diabetes, Alzheimer's, Parkinson's disease, depression, arthritis, osteoporosis, and hip fractures was self-reported.

Overall, 48 potential predictors for multimorbidity of cancer and mental disorders were included in the analysis.

Text S2. The Interaction Between the Onsets of Cancer and Mental Disorders

We used Cox proportional regression models to assess the interaction between the onsets of cancer and mental disorders. The incidence of the corresponding primary condition within seven years in all participants at baseline was considered as the reference group. For example, to examine whether cancer as the primary condition would increase the risk of mental disorders as the secondary condition, the comparison was conducted between the incidence of mental disorders as the secondary condition in participants with cancer as the primary condition, and mental disorders as the primary condition in the total population. To enable comparison, the onset of primary and secondary condition was restricted to the cases occurring within the first seven years for this

specific analysis, where participants with follow-up time <7 years for the secondary condition were excluded.

Text S3. Description of Machine Learning Method

This section describes how we applied random forest to evaluate the importance of predictors.

Factorization of Features

The data were factorized into a labeled dataset containing the independent variables (potential predictors were listed in Table 1) and the dependent variable (participants were free of 13 chronic conditions in the nine years following the baseline date) using h2o. We used the whole dataset as both training and testing data as we aimed only to obtain the variable importance metric. We applied the commonly used machine learning method random forest in the analysis.

Random Forest

Random forest is widely applied in research since its creation. The random forest algorithm is a supervised learning algorithm constructing an ensemble of decision trees using randomly bootstrapping sample datasets and averaging predictions of its trees [6]. It applies a bagging method to ensemble multiple decision trees generated from subsets to reduce correlations among the constitute decision trees. A lower correlation between decision tress is associated with a lower forest error rate. Random forest has its robustness to reduce noise and overfitting, given that the datasets are built independently using bagging method [6,7]. The strength of each individual tree in the forest is another determinist factor for the forest error rate. In this study, we used the area under curve to determine the best predicting variable and location for each tree split in our algorithm. We grew the forest with 500 trees. A five-fold cross-validation was conducted to test if the model was overfitting even though random forest is less likely to be overfitting compared with other methods [6]. We implemented grid search to obtain optimal parameters including the number of variables randomly sampled as candidates at each split and the max depth of each tree (effectively the number of interactions are considered in the model) for random forest. A range of values for each hyper-parameter was specified, and all possible combinations of the hyper-parameters were examined, while the combination with the highest cross-validation performance metric was obtained. There are several indices for the model performance, and maximization of the area under the receiver operating characteristic curve was applied in this study. For example, random forest has hyper-parameters specifying the number of trees and the max depth of each tree (effectively how many interactions are considered in the model), whereas the decision rules are the parameters.

We set the parameter nthreads as 1 as to make use of all available cores on the system.

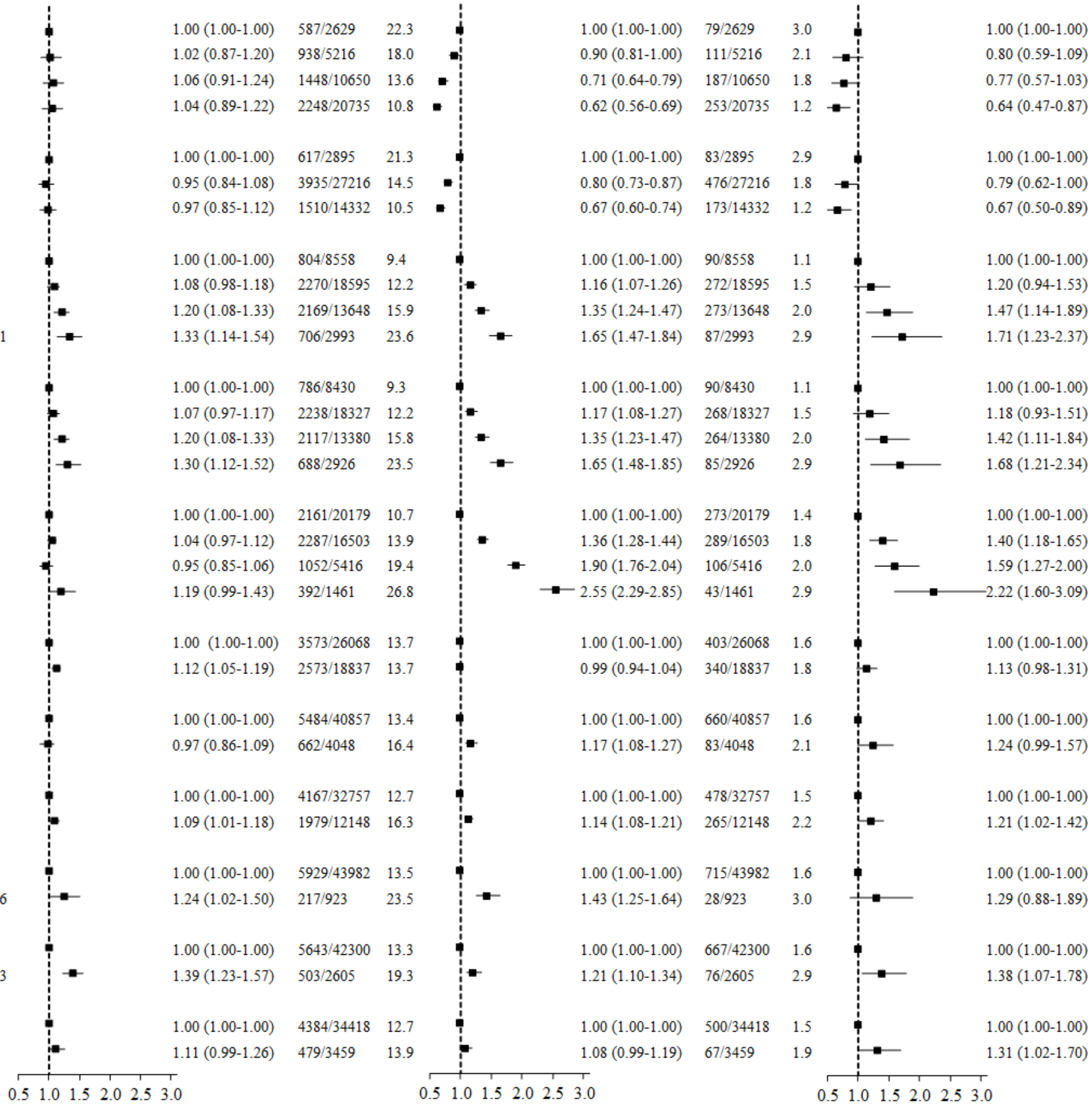

	Cancer			Mental disorders			Multimorbidity		
	Events/ participants	Incidence	HR (95% CI)	Events/ participants	Incidence	HR (95% CI)	Events/ participants	Incidence	HR (95% CI)
**Income**									
<20000 AUD	243/2629	9.2	1.00 (1.00-1.00)	587/2629	22.3	1.00 (1.00-1.00)	79/2629	3.0	1.00 (1.00-1.00)
20000-39999 AUD	473/5216	9.1	1.02 (0.87-1.20)	938/5216	18.0	0.90 (0.81-1.00)	111/5216	2.1	0.80 (0.59-1.09)
40000-69999 AUD	922/10650	8.7	1.06 (0.91-1.24)	1448/10650	13.6	0.71 (0.64-0.79)	187/10650	1.8	0.77 (0.57-1.03)
≥70000 AUD	1597/20735	7.7	1.04 (0.89-1.22)	2248/20735	10.8	0.62 (0.56-0.69)	253/20735	1.2	0.64 (0.47-0.87)
**Education level**									
<10 years	281/2895	9.7	1.00 (1.00-1.00)	617/2895	21.3	1.00 (1.00-1.00)	83/2895	2.9	1.00 (1.00-1.00)
High school/TAFE	2265/27216	8.3	0.95 (0.84-1.08)	3935/27216	14.5	0.80 (0.73-0.87)	476/27216	1.8	0.79 (0.62-1.00)
University or higher	1140/14332	8.0	0.97 (0.85-1.12)	1510/14332	10.5	0.67 (0.60-0.74)	173/14332	1.2	0.67 (0.50-0.89)
**Self-rated overal health**									
Excellent	624/8558	7.3	1.00 (1.00-1.00)	804/8558	9.4	1.00 (1.00-1.00)	90/8558	1.1	1.00 (1.00-1.00)
Very good	1495/18595	8.0	1.08 (0.98-1.18)	2270/18595	12.2	1.16 (1.07-1.26)	272/18595	1.5	1.20 (0.94-1.53)
Good	1219/13648	8.9	1.20 (1.08-1.33)	2169/13648	15.9	1.35 (1.24-1.47)	273/13648	2.0	1.47 (1.14-1.89)
Fair/Poor	302/2993	10.1	1.33 (1.14-1.54)	706/2993	23.6	1.65 (1.47-1.84)	87/2993	2.9	1.71 (1.23-2.37)
**Self-rated quality of life**									
Excellent	616/8430	7.3	1.00 (1.00-1.00)	786/8430	9.3	1.00 (1.00-1.00)	90/8430	1.1	1.00 (1.00-1.00)
Very good	1464/18327	8.0	1.07 (0.97-1.17)	2238/18327	12.2	1.17 (1.08-1.27)	268/18327	1.5	1.18 (0.93-1.51)
Good	1197/13380	9.0	1.20 (1.08-1.33)	2117/13380	15.8	1.35 (1.23-1.47)	264/13380	2.0	1.42 (1.11-1.84)
Fair/Poor	290/2926	9.9	1.30 (1.12-1.52)	688/2926	23.5	1.65 (1.48-1.85)	85/2926	2.9	1.68 (1.21-2.34)
**Psychological distress**									
Low	1708/20179	8.5	1.00 (1.00-1.00)	2161/20179	10.7	1.00 (1.00-1.00)	273/20179	1.4	1.00 (1.00-1.00)
Mild	1368/16503	8.3	1.04 (0.97-1.12)	2287/16503	13.9	1.36 (1.28-1.44)	289/16503	1.8	1.40 (1.18-1.65)
Moderate	391/5416	7.2	0.95 (0.85-1.06)	1052/5416	19.4	1.90 (1.76-2.04)	106/5416	2.0	1.59 (1.27-2.00)
High	131/1461	9.0	1.19 (0.99-1.43)	392/1461	26.8	2.55 (2.29-2.85)	43/1461	2.9	2.22 (1.60-3.09)
**Family history of cancer**									
No	2055/26068	7.9	1.00 (1.00-1.00)	3573/26068	13.7	1.00 (1.00-1.00)	403/26068	1.6	1.00 (1.00-1.00)
Yes	1679/18837	8.9	1.12 (1.05-1.19)	2573/18837	13.7	0.99 (0.94-1.04)	340/18837	1.8	1.13 (0.98-1.31)
**Family history of depression**									
No	3417/40857	8.4	1.00 (1.00-1.00)	5484/40857	13.4	1.00 (1.00-1.00)	660/40857	1.6	1.00 (1.00-1.00)
Yes	317/4048	7.8	0.97 (0.86-1.09)	662/4048	16.4	1.17 (1.08-1.27)	83/4048	2.1	1.24 (0.99-1.57)
**Hypertension**									
No	2575/32757	7.9	1.00 (1.00-1.00)	4167/32757	12.7	1.00 (1.00-1.00)	478/32757	1.5	1.00 (1.00-1.00)
Yes	1159/12148	9.5	1.09 (1.01-1.18)	1979/12148	16.3	1.14 (1.08-1.21)	265/12148	2.2	1.21 (1.02-1.42)
**Arthritis**									
No	3627/43982	8.3	1.00 (1.00-1.00)	5929/43982	13.5	1.00 (1.00-1.00)	715/43982	1.6	1.00 (1.00-1.00)
Yes	107/923	11.6	1.24 (1.02-1.50)	217/923	23.5	1.43 (1.25-1.64)	28/923	3.0	1.29 (0.88-1.89)
**Diabetes**									
No	3415/42300	8.1	1.00 (1.00-1.00)	5643/42300	13.3	1.00 (1.00-1.00)	667/42300	1.6	1.00 (1.00-1.00)
Yes	319/2605	12.3	1.39 (1.23-1.57)	503/2605	19.3	1.21 (1.10-1.34)	76/2605	2.9	1.38 (1.07-1.78)
**Asthma**									
No	2727/34418	7.9	1.00 (1.00-1.00)	4384/34418	12.7	1.00 (1.00-1.00)	500/34418	1.5	1.00 (1.00-1.00)
Yes	301/3459	8.7	1.11 (0.99-1.26)	479/3459	13.9	1.08 (0.99-1.19)	67/3459	1.9	1.31 (1.02-1.70)

Figure S1. Hazard ratios for incident cancer, mental disorders, and multimorbidity associated with age, socioeconomic status, self-rated health and psychological distress, and history and family history of chronic conditions in men. Hazard ratios were assessed using Cox regression models adjusted for age, country of birth, income, education, work status, number of children, BMI, psychological distress, hypertension, dyslipidemia, diabetes, asthma, arthritis, hip replacement, and family history of cancer, depression, heart disease, stroke, diabetes, hypertension, Parkinson’s disease, and dementia.

	Cancer			Mental disorders			Multimorbidity		
	Events/ participants	Incidence	HR (95% CI)	Events/ participants	Incidence	HR (95% CI)	Events/ participants	Incidence	HR (95% CI)
**Income**									
<20000 AUD	473/4807	9.8	1.00 (1.00-1.00)	1351/4807	28.1	1.00 (1.00-1.00)	173/4807	3.6	1.00 (1.00-1.00)
20000-39999 AUD	753/8277	9.1	0.97 (0.86-1.09)	1940/8277	23.4	0.87 (0.81-0.94)	222/8277	2.7	0.82 (0.67-1.00)
40000-69999 AUD	1046/11639	9.0	0.99 (0.89-1.11)	2259/11639	19.4	0.74 (0.69-0.80)	288/11639	2.5	0.83 (0.68-1.01)
≥70000 AUD	1550/17228	9.0	1.07 (0.95-1.20)	2738/17228	15.9	0.65 (0.61-0.70)	339/17228	2.0	0.74 (0.60-0.92)
**Education level**									
<10 years	375/3844	9.8	1.00 (1.00-1.00)	1195/3844	31.1	1.00 (1.00-1.00)	142/3844	3.7	1.00 (1.00-1.00)
High school/TAFE	3189/34197	9.3	1.01 (0.91-1.13)	7345/34197	21.5	0.76 (0.72-0.81)	920/34197	2.7	0.89 (0.74-1.07)
University or higher	1419/15516	9.2	1.02 (0.90-1.15)	2461/15516	15.9	0.63 (0.58-0.68)	319/15516	2.1	0.79 (0.64-0.99)
**Self-rated overall health**									
Excellent	1113/13411	8.3	1.00 (1.00-1.00)	1992/13411	14.9	1.00 (1.00-1.00)	222/13411	1.7	1.00 (1.00-1.00)
Very good	2097/23063	9.1	1.08 (1.00-1.16)	4427/23063	19.2	1.18 (1.12-1.25)	552/23063	2.4	1.30 (1.11-1.52)
Good	1357/13357	10.2	1.21 (1.11-1.32)	3384/13357	25.3	1.43 (1.34-1.52)	440/13357	3.3	1.62 (1.36-1.92)
Fair/Poor	300/2542	11.8	1.41 (1.22-1.62)	884/2542	34.8	1.76 (1.61-1.92)	127/2542	5.0	2.07 (1.62-2.64)
**Self-rated quality of life**									
Excellent	1090/13174	8.3	1.00 (1.00-1.00)	1951/13174	14.8	1.00 (1.00-1.00)	212/13174	1.6	1.00 (1.00-1.00)
Very good	2065/22722	9.1	1.08 (1.00-1.17)	4351/22722	19.2	1.18 (1.12-1.25)	538/22722	2.4	1.33 (1.13-1.56)
Good	1336/13101	10.2	1.22 (1.12-1.33)	3302/13101	25.2	1.42 (1.34-1.51)	431/13101	3.3	1.68 (1.41-2.01)
Fair/Poor	291/2472	11.8	1.42 (1.24-1.64)	859/2472	34.8	1.76 (1.61-1.93)	123/2472	5.0	2.17 (1.70-2.79)
**Psychological distress**									
Low	2152/22446	9.6	1.00 (1.00-1.00)	3780/22446	16.8	1.00 (1.00-1.00)	492/22446	2.2	1.00 (1.00-1.00)
Mild	1796/20063	9.0	0.99 (0.93-1.05)	4064/20063	20.3	1.30 (1.24-1.36)	492/20063	2.5	1.21 (1.07-1.37)
Moderate	627/7036	8.9	1.00 (0.92-1.10)	1912/7036	27.2	1.77 (1.67-1.87)	229/7036	3.3	1.59 (1.36-1.87)
High	188/2015	9.3	1.07 (0.92-1.25)	716/2015	35.5	2.38 (2.20-2.59)	86/2015	4.3	2.06 (1.63-2.60)
**Family history of cancer**									
No	2643/30015	8.8	1.00 (1.00-1.00)	6140/30015	20.5	1.00 (1.00-1.00)	718/30015	2.4	1.00 (1.00-1.00)
Yes	2377/24037	9.9	1.11 (1.05-1.18)	4981/24037	20.7	0.99 (0.96-1.03)	676/24037	2.8	1.15 (1.03-1.27)
**Family history of depression**									
No	4418/47203	9.4	1.00 (1.00-1.00)	9455/47203	20.0	1.00 (1.00-1.00)	1181/47203	2.5	1.00 (1.00-1.00)
Yes	602/6850	8.8	0.94 (0.86-1.02)	1666/6850	24.3	1.18 (1.12-1.25)	213/6850	3.1	1.19 (1.02-1.38)
**Hypertension**									
No	3713/42209	8.8	1.00 (1.00-1.00)	8227/42209	19.5	1.00 (1.00-1.00)	987/42209	2.3	1.00 (1.00-1.00)
Yes	1307/11844	11.0	1.16 (1.08-1.24)	2894/11844	24.4	1.15 (1.10-1.21)	407/11844	3.4	1.27 (1.12-1.44)
**Arthritis**									
No	4758/51809	9.2	1.00 (1.00-1.00)	10390/51809	20.1	1.00 (1.00-1.00)	1282/51809	2.5	1.00 (1.00-1.00)
Yes	262/2244	11.7	1.13 (1.00-1.29)	731/2244	32.6	1.48 (1.37-1.60)	112/2244	5.0	1.59 (1.30-1.93)
**Diabetes**									
No	4760/52019	9.2	1.00 (1.00-1.00)	10560/52019	20.3	1.00 (1.00-1.00)	1299/52019	2.5	1.00 (1.00-1.00)
Yes	260/2034	12.8	1.32 (1.16-1.50)	561/2034	27.6	1.21 (1.11-1.32)	95/2034	4.7	1.54 (1.23-1.91)
**Asthma**									
No	3565/40146	8.9	1.00 (1.00-1.00)	7598/40146	18.9	1.00 (1.00-1.00)	909/40146	2.3	1.00 (1.00-1.00)
Yes	489/5155	9.5	1.06 (0.96-1.16)	1192/5155	23.1	1.21 (1.14-1.29)	156/5155	3.0	1.29 (1.09-1.53)

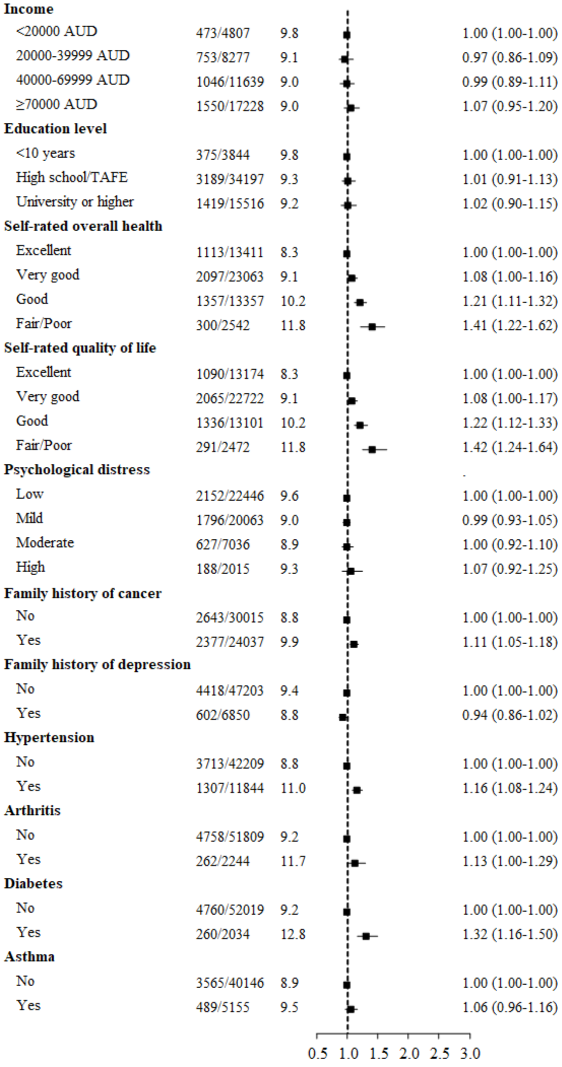

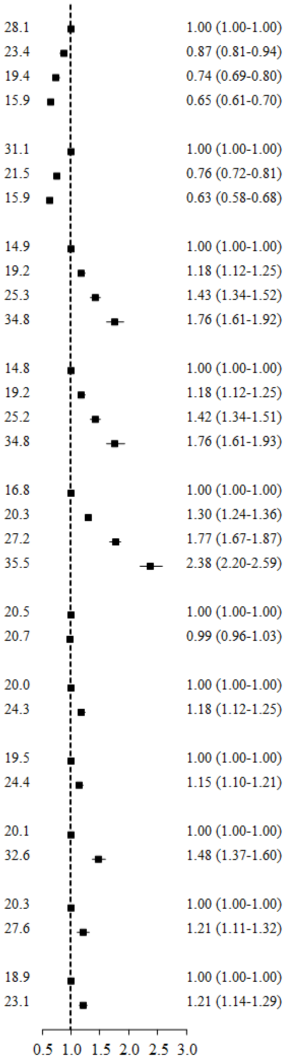

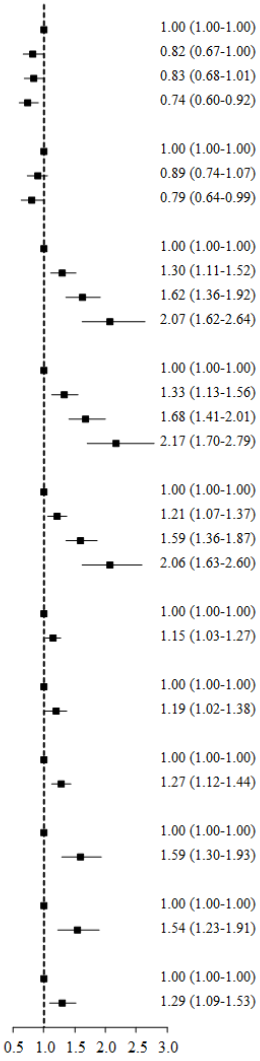

Figure S2. Hazard ratios for incident cancer, mental disorders and multimorbidity associated with age, socioeconomic status, self-rated health and psychological distress, and history and family history of chronic conditions in women. Hazard ratios were assessed using Cox regression models adjusted for age, country of birth, income, education, work status, number of children, BMI, psychological distress, hypertension, dyslipidemia, diabetes, asthma, arthritis, hip replacement, and family history of cancer, depression, heart disease, stroke, diabetes, hypertension, Parkinson's disease, and dementia.

	Cancer			Mental disorders			Multimorbidity		
	Events/ participants	Incidence	HR (95% CI)	Events/ participants	Incidence	HR (95% CI)	Events/ participants	Incidence	HR (95% CI)
**Smoking**									
Never	1857/24683	7.5	1.00 (1.00-1.00)	2799/24683	11.3	1.00 (1.00-1.00)	298/24683	1.2	1.00 (1.00-1.00)
Former	1465/16201	9.0	1.13 (1.05-1.21)	2537/16201	15.7	1.26 (1.19-1.33)	334/16201	2.1	1.44 (1.23-1.68)
Current	411/4011	10.3	1.43 (1.28-1.60)	809/4011	20.2	1.53 (1.41-1.66)	111/4011	2.8	2.06 (1.64-2.58)
**Sleep duration**									
<7 or >9 hours/day	696/8483	8.2	1.00 (1.00-1.00)	1374/8483	16.2	1.00 (1.00-1.00)	149/8483	1.8	1.00 (1.00-1.00)
7-9 hours/day	2969/35582	8.3	1.03 (0.95-1.12)	4613/35582	13.0	0.90 (0.84-0.95)	575/35582	1.6	1.04 (0.87-1.25)
**Vegetables intake**									
0 or 1 serving/day	612/7135	8.6	1.00 (1.00-1.00)	1098/7135	15.4	1.00 (1.00-1.00)	123/7135	1.7	1.00 (1.00-1.00)
2 servings/day	1184/14748	8.0	0.96 (0.87-1.06)	1980/14748	13.4	0.92 (0.85-0.99)	242/14748	1.6	1.02 (0.82-1.27)
3 servings/day	582/7116	8.2	0.98 (0.87-1.09)	904/7116	12.7	0.88 (0.81-0.96)	115/7116	1.6	1.02 (0.79-1.32)
4 servings/day	480/5571	8.6	1.01 (0.90-1.14)	678/5571	12.2	0.83 (0.75-0.91)	69/5571	1.2	0.76 (0.56-1.02)
≥5 servings/day	779/9313	8.4	0.95 (0.85-1.06)	1302/9313	14.0	0.92 (0.85-1.00)	170/9313	1.8	1.05 (0.83-1.32)
**Fruit intake**									
None	386/4373	8.8	1.00 (1.00-1.00)	695/4373	15.9	1.00 (1.00-1.00)	86/4373	2.0	1.00 (1.00-1.00)
1 serving/day	1411/16656	8.5	0.94 (0.84-1.05)	2283/16656	13.7	0.92 (0.84-1.00)	274/16656	1.7	0.86 (0.67-1.10)
2 servings/day	996/12268	8.1	0.89 (0.79-1.00)	1592/12268	13.0	0.88 (0.81-0.97)	205/12268	1.7	0.88 (0.68-1.14)
≥3 servings/day	724/9066	8.0	0.87 (0.77-0.98)	1111/9066	12.3	0.83 (0.76-0.92)	123/9066	1.4	0.71 (0.54-0.94)
**Chicken intake**									
None	108/1509	7.2	1.00 (1.00-1.00)	177/1509	11.7	1.00 (1.00-1.00)	28/1509	1.9	1.00 (1.00-1.00)
1 serving/week	674/7950	8.5	1.17 (0.95-1.44)	1054/7950	13.3	1.12 (0.95-1.32)	134/7950	1.7	0.87 (0.58-1.32)
2 servings/week	1062/13208	8.0	1.16 (0.95-1.41)	1658/13208	12.6	1.09 (0.93-1.27)	200/13208	1.5	0.84 (0.56-1.26)
≥3 servings/week	1078/13881	7.8	1.15 (0.95-1.41)	1749/13881	12.6	1.12 (0.96-1.31)	187/13881	1.4	0.80 (0.53-1.19)
**Physical activity**									
0-4 sessions/week	717/8094	8.9	1.00 (1.00-1.00)	1148/8094	14.2	1.00 (1.00-1.00)	142/8094	1.8	1.00 (1.00-1.00)
5-9 sessions/week	980/12242	8.0	0.89 (0.81-0.98)	1668/12242	13.6	1.00 (0.92-1.08)	202/12242	1.7	0.96 (0.78-1.19)
10-14 sessions/week	819/9805	8.4	0.93 (0.84-1.03)	1337/9805	13.6	1.02 (0.94-1.10)	148/9805	1.5	0.89 (0.70-1.12)
≥15 sessions/week	1120/13552	8.3	0.92 (0.84-1.01)	1775/13552	13.1	0.97 (0.90-1.04)	224/13552	1.7	0.98 (0.79-1.21)
**Body mass index**									
15-24.9 kg/m^2	989/12508	7.9	1.00 (1.00-1.00)	1514/12508	12.1	1.00 (1.00-1.00)	169/12508	1.4	1.00 (1.00-1.00)
25-29.9 kg/m^2	1666/20591	8.1	0.98 (0.91-1.07)	2724/20591	13.2	1.07 (1.00-1.14)	326/20591	1.6	1.09 (0.90-1.31)
≥30 kg/m^2	893/9667	9.2	1.09 (0.99-1.20)	1595/9667	16.5	1.22 (1.13-1.31)	213/9667	2.2	1.33 (1.07-1.65)

Figure S3. Hazard ratios for incident cancer, mental disorders, and multimorbidity associated with behavioral factors in men. Hazard ratios were assessed using Cox regression models adjusted for age, country of birth, income, education, work status, number of children, BMI, psychological distress, hypertension, dyslipidemia, diabetes, asthma, arthritis, hip replacement, and family history of cancer, depression, heart disease, stroke, diabetes, hypertension, Parkinson’s disease, and dementia.

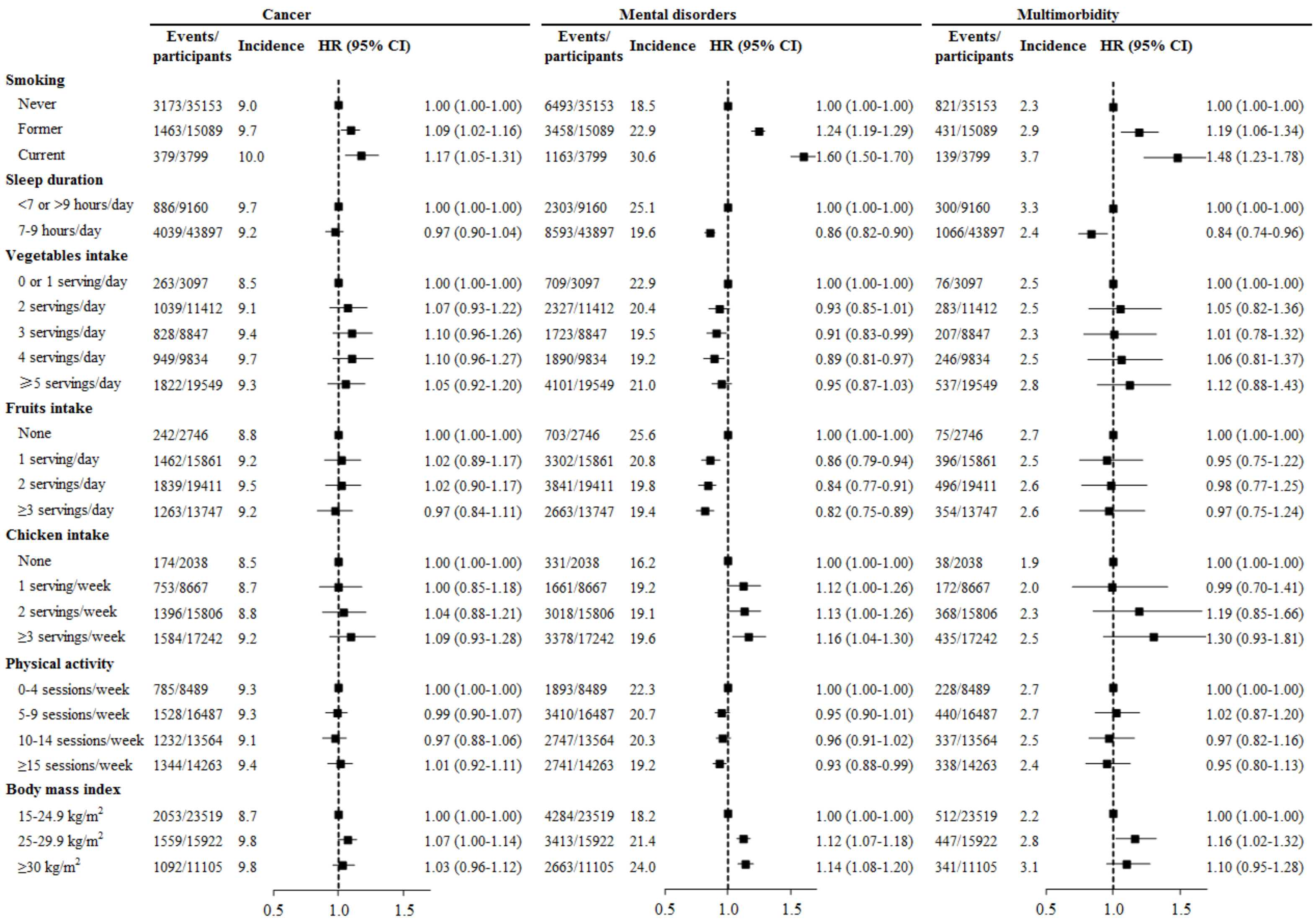

Cancer
Mental disorders
Multimorbidity
Events/ participants
Incidence
HR (95% CI)
Smoking
Never 3173/35153 9.0 1.00 (1.00-1.00) 6493/35153 18.5 1.00 (1.00-1.00) 821/35153 2.3 1.00 (1.00-1.00)
Former 1463/15089 9.7 1.09 (1.02-1.16) 3458/15089 22.9 1.24 (1.19-1.29) 431/15089 2.9 1.19 (1.06-1.34)
Current 379/3799 10.0 1.17 (1.05-1.31) 1163/3799 30.6 1.60 (1.50-1.70) 139/3799 3.7 1.48 (1.23-1.78)
Sleep duration
<7 or >9 hours/day 886/9160 9.7 1.00 (1.00-1.00) 2303/9160 25.1 1.00 (1.00-1.00) 300/9160 3.3 1.00 (1.00-1.00)
7-9 hours/day 4039/43897 9.2 0.97 (0.90-1.04) 8593/43897 19.6 0.86 (0.82-0.90) 1066/43897 2.4 0.84 (0.74-0.96)
Vegetables intake
0 or 1 serving/day 263/3097 8.5 1.00 (1.00-1.00) 709/3097 22.9 1.00 (1.00-1.00) 76/3097 2.5 1.00 (1.00-1.00)
2 servings/day 1039/11412 9.1 1.07 (0.93-1.22) 2327/11412 20.4 0.93 (0.85-1.01) 283/11412 2.5 1.05 (0.82-1.36)
3 servings/day 828/8847 9.4 1.10 (0.96-1.26) 1723/8847 19.5 0.91 (0.83-0.99) 207/8847 2.3 1.01 (0.78-1.32)
4 servings/day 949/9834 9.7 1.10 (0.96-1.27) 1890/9834 19.2 0.89 (0.81-0.97) 246/9834 2.5 1.06 (0.81-1.37)
≥5 servings/day 1822/19549 9.3 1.05 (0.92-1.20) 4101/19549 21.0 0.95 (0.87-1.03) 537/19549 2.8 1.12 (0.88-1.43)
Fruits intake
None 242/2746 8.8 1.00 (1.00-1.00) 703/2746 25.6 1.00 (1.00-1.00) 75/2746 2.7 1.00 (1.00-1.00)
1 serving/day 1462/15861 9.2 1.02 (0.89-1.17) 3302/15861 20.8 0.86 (0.79-0.94) 396/15861 2.5 0.95 (0.75-1.22)
2 servings/day 1839/19411 9.5 1.02 (0.90-1.17) 3841/19411 19.8 0.84 (0.77-0.91) 496/19411 2.6 0.98 (0.77-1.25)
≥3 servings/day 1263/13747 9.2 0.97 (0.84-1.11) 2663/13747 19.4 0.82 (0.75-0.89) 354/13747 2.6 0.97 (0.75-1.24)
Chicken intake
None 174/2038 8.5 1.00 (1.00-1.00) 331/2038 16.2 1.00 (1.00-1.00) 38/2038 1.9 1.00 (1.00-1.00)
1 serving/week 753/8667 8.7 1.00 (0.85-1.18) 1661/8667 19.2 1.12 (1.00-1.26) 172/8667 2.0 0.99 (0.70-1.41)
2 servings/week 1396/15806 8.8 1.04 (0.88-1.21) 3018/15806 19.1 1.13 (1.00-1.26) 368/15806 2.3 1.19 (0.85-1.66)
≥3 servings/week 1584/17242 9.2 1.09 (0.93-1.28) 3378/17242 19.6 1.16 (1.04-1.30) 435/17242 2.5 1.30 (0.93-1.81)
Physical activity
0-4 sessions/week 785/8489 9.3 1.00 (1.00-1.00) 1893/8489 22.3 1.00 (1.00-1.00) 228/8489 2.7 1.00 (1.00-1.00)
5-9 sessions/week 1528/16487 9.3 0.99 (0.90-1.07) 3410/16487 20.7 0.95 (0.90-1.01) 440/16487 2.7 1.02 (0.87-1.20)
10-14 sessions/week 1232/13564 9.1 0.97 (0.88-1.06) 2747/13564 20.3 0.96 (0.91-1.02) 337/13564 2.5 0.97 (0.82-1.16)
≥15 sessions/week 1344/14263 9.4 1.01 (0.92-1.11) 2741/14263 19.2 0.93 (0.88-0.99) 338/14263 2.4 0.95 (0.80-1.13)
Body mass index
15-24.9 kg/m^2 2053/23519 8.7 1.00 (1.00-1.00) 4284/23519 18.2 1.00 (1.00-1.00) 512/23519 2.2 1.00 (1.00-1.00)
25-29.9 kg/m^2 1559/15922 9.8 1.07 (1.00-1.14) 3413/15922 21.4 1.12 (1.07-1.18) 447/15922 2.8 1.16 (1.02-1.32)
≥30 kg/m^2 1092/11105 9.8 1.03 (0.96-1.12) 2663/11105 24.0 1.14 (1.08-1.20) 341/11105 3.1 1.10 (0.95-1.28)
0.5 1.0 1.5
0.5 1.0 1.5
0.5 1.0 1.5

Figure S4. Hazard ratios for incident cancer, mental disorders, and multimorbidity associated with behavioral factors in women. Hazard ratios were assessed using Cox regression models adjusted for age, country of birth, income, education, work status, number of children, BMI, psychological distress, hypertension, dyslipidemia, diabetes, asthma, arthritis, hip replacement, and family history of cancer, depression, heart disease, stroke, diabetes, hypertension, Parkinson's disease, and dementia.

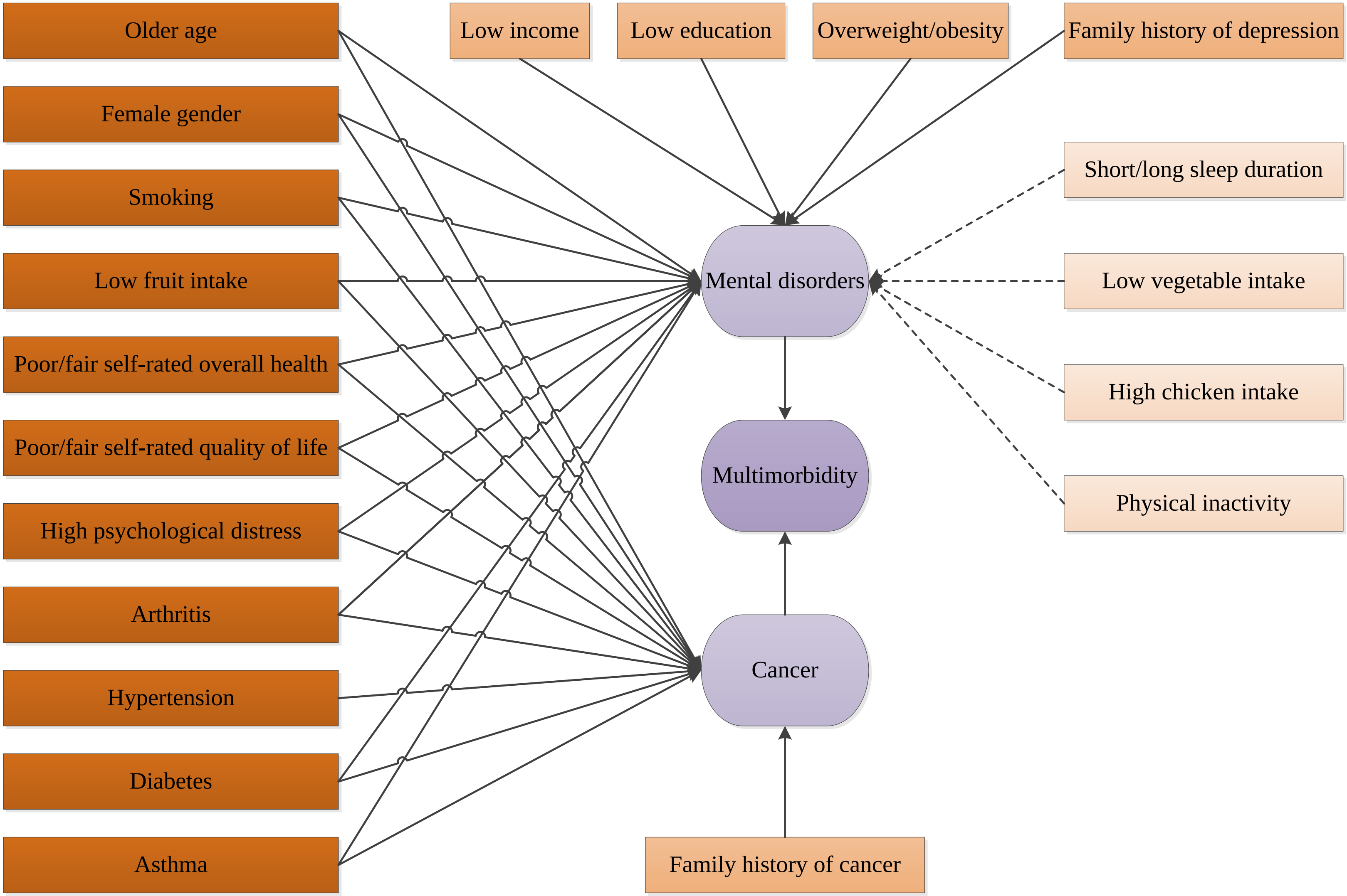
Older age
Female gender
Smoking
Low fruit intake
Poor/fair self-rated overall health
Poor/fair self-rated quality of life
High psychological distress
Arthritis
Hypertension
Diabetes
Asthma
Low income
Low education
Overweight/obesity
Family history of depression
Short/long sleep duration
Low vegetable intake
High chicken intake
Physical inactivity
Mental disorders
Multimorbidity
Cancer
Family history of cancer

Figure S5. Classification of risk factors for incident cancer, mental disorders, and multimorbidity. The risk factors were categorized into four groups according to their relationships with cancer, mental disorders, and multimorbidity presented in Figure 1–4 and Figure S2–S7. Older age, female gender, smoking, low fruit intake, poor/fair self-rated health, poor/fair self-rated quality of life, high psychological distress, hypertension, arthritis, asthma, and diabetes were classified as shared risk factors for cancer and mental disorders as they were associated with an increased risk of incident cancer, mental disorders, and multimorbidity. Low education, low income, overweight/obesity, and family history of depression were classified as risk factors for mental disorders given that they were associated with an increased risk of incident mental disorders but not incident cancer, and their positive association with multimorbidity might depend on mental disorders. Family history of cancer was classified as a risk factor for cancer given that it was associated with an increased risk of incident cancer but not incident mental disorders and its positive association with multimorbidity might depend on cancer. Long/short sleep duration, low vegetable intake, high chicken intake, and physical inactivity were classified as risk factors for mental disorders only since they were associated with an increased risk of incident mental disorders but not incident cancer or multimorbidity.

	Cancer			Depression			Multimorbidity		
	Events/ participants	Incidence	HR (95% CI)	Events/ participants	Incidence	HR (95% CI)	Events/ participants	Incidence	HR (95% CI)
**Income**									
<20000 AUD	723/7485	9.7	1.00 (1.00-1.00)	1564/7485	20.9	1.00 (1.00-1.00)	200/7485	2.7	1.00 (1.00-1.00)
20000-39999 AUD	1210/13471	9.0	0.98 (0.89-1.08)	2295/13471	17.0	0.89 (0.83-0.95)	268/13471	2.0	0.86 (0.71-1.04)
40000-69999 AUD	1956/22244	8.8	1.02 (0.94-1.12)	2875/22244	12.9	0.73 (0.68-0.78)	390/22244	1.8	0.89 (0.74-1.07)
≥70000 AUD	3141/37911	8.3	1.05 (0.96-1.15)	3809/37911	10.1	0.64 (0.59-0.68)	476/37911	1.3	0.78 (0.65-0.95)
**Education level**									
<10 years	652/6730	9.7	1.00 (1.00-1.00)	1488/6730	22.1	1.00 (1.00-1.00)	185/6730	2.8	1.00 (1.00-1.00)
High school/TAFE	5421/61335	8.8	0.98 (0.90-1.06)	8841/61335	14.4	0.76 (0.71-0.80)	1127/61335	1.8	0.82 (0.70-0.96)
University or higher	2556/29830	8.6	0.99 (0.91-1.09)	3002/29830	10.1	0.61 (0.57-0.65)	376/29830	1.3	0.67 (0.55-0.81)
**Self-rated overall health**									
Excellent	1736/21974	7.9	1.00 (1.00-1.00)	2103/21974	9.6	1.00 (1.00-1.00)	245/21974	1.1	1.00 (1.00-1.00)
Very good	3580/41654	8.6	1.07 (1.01-1.14)	5179/41654	12.4	1.18 (1.12-1.25)	658/41654	1.6	1.27 (1.09-1.47)
Good	2565/26962	9.5	1.20 (1.13-1.28)	4398/26962	16.3	1.40 (1.33-1.49)	575/26962	2.1	1.56 (1.33-1.83)
Fair/Poor	588/5489	10.7	1.35 (1.22-1.50)	1312/5489	23.9	1.77 (1.64-1.91)	170/5489	3.1	1.90 (1.53-2.37)
**Self-rated quality of life**									
Excellent	1705/21611	7.9	1.00 (1.00-1.00)	2056/21611	9.5	1.00 (1.00-1.00)	237/21611	1.1	1.00 (1.00-1.00)
Very good	3517/41046	8.6	1.07 (1.01-1.14)	5090/41046	12.4	1.19 (1.13-1.25)	644/41046	1.6	1.28 (1.10-1.49)
Good	2521/26440	9.5	1.21 (1.13-1.29)	4287/26440	16.2	1.40 (1.32-1.48)	559/26440	2.1	1.58 (1.34-1.86)
Fair/Poor	570/5351	10.7	1.36 (1.22-1.51)	1271/5351	23.8	1.77 (1.63-1.91)	166/5351	3.1	1.97 (1.58-2.45)
**Psychological distress**									
Low	3837/42511	9.0	1.00 (1.00-1.00)	4349/42511	10.2	1.00 (1.00-1.00)	591/42511	1.4	1.00 (1.00-1.00)
Mild	3157/36503	8.7	1.02 (0.97-1.06)	5034/36503	13.8	1.42 (1.37-1.48)	642/36503	1.8	1.36 (1.22-1.53)
Moderate	1019/12499	8.2	0.98 (0.92-1.05)	2435/12499	19.5	1.99 (1.90-2.10)	273/12499	2.2	1.67 (1.45-1.94)
High	322/3499	9.2	1.13 (1.01-1.27)	972/3499	27.8	2.84 (2.64-3.05)	109/3499	3.1	2.29 (1.86-2.82)
**Family history of cancer**									
No	4671/56038	8.3	1.00 (1.00-1.00)	7621/56038	13.6	1.00 (1.00-1.00)	884/56038	1.6	1.00 (1.00-1.00)
Yes	4042/42814	9.4	1.12 (1.07-1.17)	5884/42814	13.7	0.99 (0.95-1.02)	823/42814	1.9	1.17 (1.06-1.29)
**Family history of depression**									
No	7792/87952	8.9	1.00 (1.00-1.00)	11653/87952	13.3	1.00 (1.00-1.00)	1468/87952	1.7	1.00 (1.00-1.00)
Yes	921/10901	8.5	0.95 (0.89-1.02)	1852/10901	17.0	1.17 (1.11-1.23)	239/10901	2.2	1.20 (1.04-1.38)
**Hypertension**									
No	6272/75072	8.4	1.00 (1.00-1.00)	9749/75072	13.0	1.00 (1.00-1.00)	1180/75072	1.6	1.00 (1.00-1.00)
Yes	2441/23781	10.3	1.13 (1.08-1.19)	3756/23781	15.8	1.47 (1.37-1.59)	527/23781	2.2	1.20 (1.07-1.35)
**Arthritis**									
No	8338/95665	8.7	1.00 (1.00-1.00)	12737/95665	13.3	1.00 (1.00-1.00)	1583/95665	1.7	1.00 (1.00-1.00)
Yes	375/3188	11.8	1.17 (1.05-1.30)	768/3188	24.1	1.14 (1.09-1.19)	124/3188	3.9	1.65 (1.37-1.99)
**Diabetes**									
No	8154/94392	8.6	1.00 (1.00-1.00)	12686/94392	13.4	1.00 (1.00-1.00)	1570/94392	1.7	1.00 (1.00-1.00)
Yes	559/4461	12.5	1.39 (1.27-1.52)	819/4461	18.4	1.24 (1.15-1.33)	137/4461	3.1	1.57 (1.30-1.89)
**Asthma**									
No	6261/74441	8.4	1.00 (1.00-1.00)	9264/74441	12.4	1.00 (1.00-1.00)	1116/74441	1.5	1.00 (1.00-1.00)
Yes	780/8604	9.1	1.06 (0.99-1.15)	1286/8604	15.0	1.15 (1.08-1.22)	174/8604	2.0	1.26 (1.07-1.48)

0.5 1.0 1.5 2.0 2.5 3.0

Figure S6. Hazard ratios for incident cancer, depression, and multimorbidity associated with age, socioeconomic status, self-rated health and psychological distress, and history and family history of chronic conditions. Hazard ratios were assessed using Cox regression models adjusted for age, gender, country of birth, income, education, work status, number of children, BMI, psychological distress, hypertension, dyslipidemia, diabetes, asthma, arthritis, hip replacement, and family history of cancer, depression, heart disease, stroke, diabetes, hypertension, Parkinson's disease, and dementia.

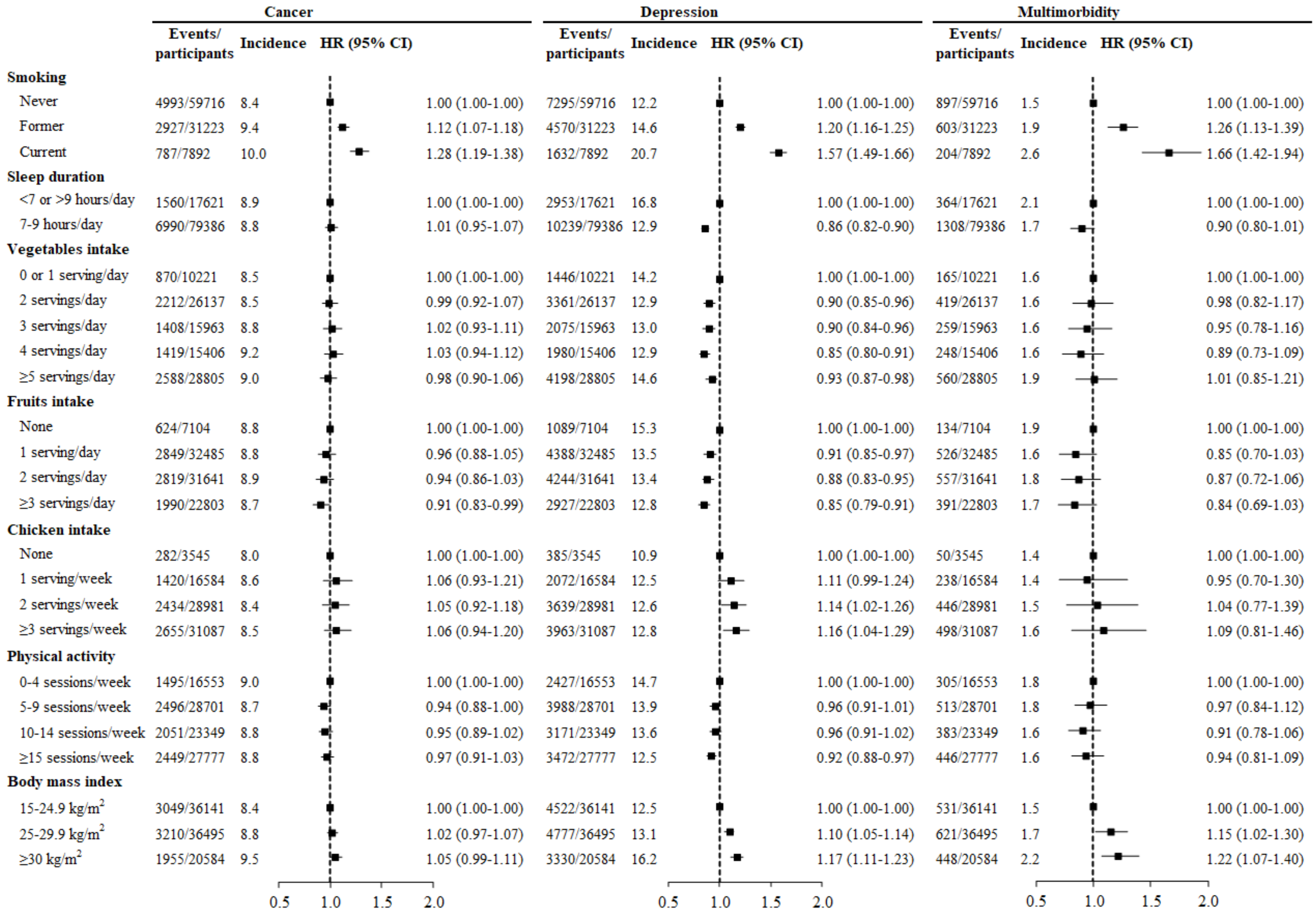

	Cancer Events/ participants	Cancer Incidence	Cancer HR (95% CI)	Depression Events/ participants	Depression Incidence	Depression HR (95% CI)	Multimorbidity Events/ participants	Multimorbidity Incidence	Multimorbidity HR (95% CI)
**Smoking**									
Never	4993/59716	8.4	1.00 (1.00-1.00)	7295/59716	12.2	1.00 (1.00-1.00)	897/59716	1.5	1.00 (1.00-1.00)
Former	2927/31223	9.4	1.12 (1.07-1.18)	4570/31223	14.6	1.20 (1.16-1.25)	603/31223	1.9	1.26 (1.13-1.39)
Current	787/7892	10.0	1.28 (1.19-1.38)	1632/7892	20.7	1.57 (1.49-1.66)	204/7892	2.6	1.66 (1.42-1.94)
**Sleep duration**									
<7 or >9 hours/day	1560/17621	8.9	1.00 (1.00-1.00)	2953/17621	16.8	1.00 (1.00-1.00)	364/17621	2.1	1.00 (1.00-1.00)
7-9 hours/day	6990/79386	8.8	1.01 (0.95-1.07)	10239/79386	12.9	0.86 (0.82-0.90)	1308/79386	1.7	0.90 (0.80-1.01)
**Vegetables intake**									
0 or 1 serving/day	870/10221	8.5	1.00 (1.00-1.00)	1446/10221	14.2	1.00 (1.00-1.00)	165/10221	1.6	1.00 (1.00-1.00)
2 servings/day	2212/26137	8.5	0.99 (0.92-1.07)	3361/26137	12.9	0.90 (0.85-0.96)	419/26137	1.6	0.98 (0.82-1.17)
3 servings/day	1408/15963	8.8	1.02 (0.93-1.11)	2075/15963	13.0	0.90 (0.84-0.96)	259/15963	1.6	0.95 (0.78-1.16)
4 servings/day	1419/15406	9.2	1.03 (0.94-1.12)	1980/15406	12.9	0.85 (0.80-0.91)	248/15406	1.6	0.89 (0.73-1.09)
≥5 servings/day	2588/28805	9.0	0.98 (0.90-1.06)	4198/28805	14.6	0.93 (0.87-0.98)	560/28805	1.9	1.01 (0.85-1.21)
**Fruits intake**									
None	624/7104	8.8	1.00 (1.00-1.00)	1089/7104	15.3	1.00 (1.00-1.00)	134/7104	1.9	1.00 (1.00-1.00)
1 serving/day	2849/32485	8.8	0.96 (0.88-1.05)	4388/32485	13.5	0.91 (0.85-0.97)	526/32485	1.6	0.85 (0.70-1.03)
2 servings/day	2819/31641	8.9	0.94 (0.86-1.03)	4244/31641	13.4	0.88 (0.83-0.95)	557/31641	1.8	0.87 (0.72-1.06)
≥3 servings/day	1990/22803	8.7	0.91 (0.83-0.99)	2927/22803	12.8	0.85 (0.79-0.91)	391/22803	1.7	0.84 (0.69-1.03)
**Chicken intake**									
None	282/3545	8.0	1.00 (1.00-1.00)	385/3545	10.9	1.00 (1.00-1.00)	50/3545	1.4	1.00 (1.00-1.00)
1 serving/week	1420/16584	8.6	1.06 (0.93-1.21)	2072/16584	12.5	1.11 (0.99-1.24)	238/16584	1.4	0.95 (0.70-1.30)
2 servings/week	2434/28981	8.4	1.05 (0.92-1.18)	3639/28981	12.6	1.14 (1.02-1.26)	446/28981	1.5	1.04 (0.77-1.39)
≥3 servings/week	2655/31087	8.5	1.06 (0.94-1.20)	3963/31087	12.8	1.16 (1.04-1.29)	498/31087	1.6	1.09 (0.81-1.46)
**Physical activity**									
0-4 sessions/week	1495/16553	9.0	1.00 (1.00-1.00)	2427/16553	14.7	1.00 (1.00-1.00)	305/16553	1.8	1.00 (1.00-1.00)
5-9 sessions/week	2496/28701	8.7	0.94 (0.88-1.00)	3988/28701	13.9	0.96 (0.91-1.01)	513/28701	1.8	0.97 (0.84-1.12)
10-14 sessions/week	2051/23349	8.8	0.95 (0.89-1.02)	3171/23349	13.6	0.96 (0.91-1.02)	383/23349	1.6	0.91 (0.78-1.06)
≥15 sessions/week	2449/27777	8.8	0.97 (0.91-1.03)	3472/27777	12.5	0.92 (0.88-0.97)	446/27777	1.6	0.94 (0.81-1.09)
**Body mass index**									
15-24.9 kg/m^2	3049/36141	8.4	1.00 (1.00-1.00)	4522/36141	12.5	1.00 (1.00-1.00)	531/36141	1.5	1.00 (1.00-1.00)
25-29.9 kg/m^2	3210/36495	8.8	1.02 (0.97-1.07)	4777/36495	13.1	1.10 (1.05-1.14)	621/36495	1.7	1.15 (1.02-1.30)
≥30 kg/m^2	1955/20584	9.5	1.05 (0.99-1.11)	3330/20584	16.2	1.17 (1.11-1.23)	448/20584	2.2	1.22 (1.07-1.40)

Figure S7. Hazard ratios for incident cancer, depression, and multimorbidity associated with behavioral factors. Hazard ratios were assessed using Cox regression models adjusted for age, gender, country of birth, income, education, work status, number of children, BMI, psychological distress, hypertension, dyslipidemia, diabetes, asthma, arthritis, hip replacement, and family history of cancer, depression, heart disease, stroke, diabetes, hypertension, Parkinson's disease, and dementia.

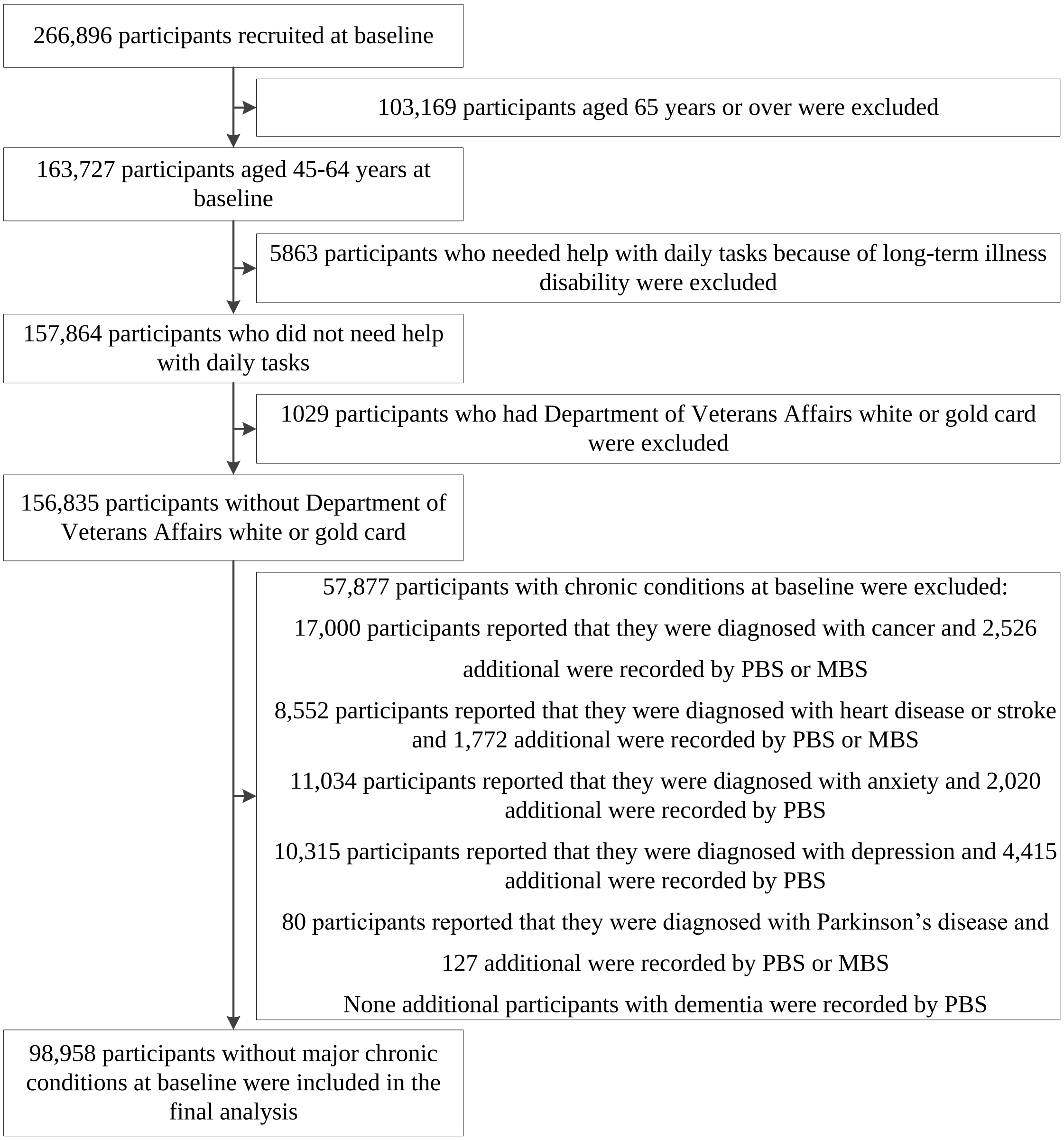

Figure S8. Flowchart of participant selection from the 45 and Up Study for the analysis in this study.

Table S1. List for Pharmaceutical Benefits Scheme and Medicare Benefits Schedule codes[†].

Chronic Conditions	Pharmaceutical Benefits Scheme Codes	ATC Codes for Pharmaceutical Benefits Scheme	Medicare Benefits Schedule Codes	Corresponding ICD Codes
Cancer[*,†]	1031G, 1079T, 1080W, 1134Q, 1144F, 1145G, 1160C, 1161D, 1162E, 1164G, 1265N, 1336H, 1340M, 1342P, 1390E, 1811H, 1929M, 1930N, 1931P, 1932Q, 2198Q, 2199R, 2315W, 2371T, 2372W, 2374Y, 2381H, 2521Q, 2528C, 2548D, 2561T, 2578Q, 2579R, 2580T, 2581W, 2582X, 2583Y, 2585C, 2884T, 2885W, 2904W, 2910E, 3017T, 3026G, 4222F, 4223G, 4309T, 4319H, 4326Q, 4327R, 4357H, 4360L, 4361M, 4364Q, 4394G, 4402Q, 4403R, 4428C, 4429D, 4431F, 4433H, 4439P, 4448D, 4451G, 4502Y, 4512L, 4514N, 4531L, 4567J, 4600D, 4610P, 4613T, 4614W, 4615X, 4618C, 4619D, 4620E, 4632T, 4639E, 4650R, 4703M, 4706Q, 4712B, 4713C, 4725Q, 4732C, 5149B, 5156J, 5270J, 5271K, 5272L, 5273M, 5274N, 5275P, 5428Q, 5429R, 5430T, 5431W, 5432X, 5433Y, 5462L, 5463M, 5464N, 5485Q, 5486R, 5487T, 5488W, 5489X, 5581R, 5582T, 5583W, 5584X, 5585Y, 5586B, 5587C, 5588D, 5589E, 5590F, 5591G, 5592H, 5593J, 5594K, 5595L, 5596M, 5597N, 5598P, 5705G, 5801H, 5804L, 5807P, 5808Q, 5809R, 5810T, 5811W, 5812X, 5813Y, 5814B, 5833B, 5834C, 5835D, 5842L, 5843M, 5844N, 5845P, 5846Q, 5847R, 5852B, 5854D, 5855E, 5856F, 5859J, 5860K, 5861L, 5862M, 5864P, 5865Q, 5866R, 5867T, 5868W, 5869X, 5872C, 5873D, 5874E, 5875F, 5876G, 5879K, 5880L, 5881M, 5882N, 5883P, 5887W, 5889Y, 5891C, 5892D, 5896H, 5897J, 5903Q, 5906W, 5907X, 5908Y, 5909B, 5910C, 5911D, 5912E, 5914G, 5915H, 5916J, 5917K, 5918L, 5919M, 5920N, 5921P, 5922Q, 5925W, 5926X, 5927Y, 5931E, 5932F, 5933G, 5934H, 5935J, 5936K, 5937L, 5943T, 5944W, 5957M, 5958N, 5959P, 5962T, 5963W, 5964X,	L01AA01, L01AA02, L01AA03, L01AA06, L01AB01, L01AX03, L01BA01, L01BA03, L01BA04, L01BB02, L01BB03, L01BB04, L01BC01, L01BC02, L01BC05, L01BC06, L01CA01, L01CA02, L01CA04, L01CB01, L01CD01, L01CD02, L01DB01, L01DB07, L01DC01, L01XA01, L01XA02, L01XC02, L01XC03, L01XE01, L01XE06, L01XE07, L01XX05, L01XX19, L01XX32	32036, 32099, 32102, 32103, 32104, 32106, 32108, 30299, 30300, 30301, 30302, 30303, 42801, 42802, 42803, 42805, 42807, 42809, 31340, 52036, 52039, 52048, 52045, 52042, 31372, 31373, 31374, 31375, 31376, 37227, 35720, 13915, 13918, 13921, 13924, 13927, 13930, 13933, 13936, 13939, 13942, 13945, 13948, 15000, 15003, 15006, 15009, 15012, 15100, 15103, 15106, 15109, 15112, 15115, 15211, 15214, 15215, 15218, 15221, 15224, 15227, 15230, 15233, 15236, 15239, 15242, 15245, 15248, 15251, 15254, 15257, 15260, 15263, 15266, 15269, 15272, 15275, 15303, 15304, 15307, 15308, 15311, 15312, 15315, 15316, 15319, 15320, 15323, 15324, 15327, 15328, 15331, 15332, 15335, 15336, 15339, 15342, 15345, 15348, 15351, 15354, 15357, 15600, 15700, 15705, 15710, 15715, 15900, 16003, 16006, 16009, 16012, 16015, 16018	C00-C97 (excluding C44)

5965Y, 5966B, 5973J, 5974K, 5975L, 5976M, 5977N, 5978P, 5979Q, 5980R, 5981T, 5982W, 5983X, 5988E, 5989F, 5990G, 5991H, 5992J, 5993K, 6007E, 6008F, 6009G, 6010H, 6249X, 6440Y, 6441B, 6444E, 6445F, 6446G, 6447H, 6497Y, 6687Y, 6688B, 6689C, 6690D, 6691E, 6692F, 6693G, 6694H, 6695J, 6696K, 6697L, 6698M, 6699N, 6700P, 6701Q, 6702R, 6703T, 6704W, 6705X, 6706Y, 6707B, 6708C, 6709D, 6710E, 6711F, 6713H, 6714J, 6716L, 6843E, 6844F, 6845G, 6846H, 6847J, 6848K, 6891Q, 6892R, 6893T, 6894W, 6895X, 6896Y, 7050C, 7051D, 7052E, 7053F, 7054G, 7055H, 7086Y, 7087B, 7088C, 7089D, 7222D, 7224F, 7225G, 7226H, 7227J, 7228K, 7229L, 7230M, 7234R, 7235T, 7237X, 7238Y, 7239B, 7244G, 7246J, 7248L, 7249M, 7250N, 7251P, 7252Q, 7254T, 7255W, 7256X, 7257Y, 7258B, 7259C, 7261E, 7262F, 7263G, 7264H, 7265J, 7266K, 7267L, 7268M, 7269N, 7270P, 7271Q, 7272R, 7274W, 7275X, 7281F, 7282G, 7283H, 7284J, 7285K, 8018B, 8033T, 8034W, 8049P, 8050Q, 8071T, 8074Y, 8076C, 8077D, 8120J, 8280T, 8281W, 8284B, 8293L, 8294M, 8360B, 8414W, 8415X, 8515E, 8569B, 8570C, 8665C, 8666D, 8800E, 8809P, 8827N, 8828P, 8850T, 8851W, 8852X, 8863L, 8967Y, 8986Y, 8987B, 8988C, 8989D, 8990E, 8991F, 8992G, 8995K, 8996L, 9005Y, 9117W, 9118X, 9119Y, 9130M, 9131N, 9282M, 9283N, 9284P, 9291B, 9341P, 9401T, 9402W, 9410G, 9414L, 9415M, 9463C, 9689Y, 9690B, 9691C, 9713F, 9729C, 10148D, 10150F, 10158P, 10165B, 10179R, 10193L, 10269L, 10270M, 10296X, 10324J, 10346M, 10362J, 10381J, 10383L, 10391X, 10401K, 10402L, 10423N, 10575N, 10576P, 10581X, 10583B, 10588G, 10589H, 10591K, 10593M, 10595P, 10597R, 10708N, 10710Q, 10720F, 10741H, 10743K,

	10744L, 10811B, 10817H, 10829Y			
Depression†	2418G, 2429W, 2417F, 1561E, 1358L, 1357K, 1012G, 1011F, 1013H, 2420J, 2421K, 2523T, 2522R, 8702B, 8703C, 8220P, 8700X, 8701Y, 10181W, 8270G, 1434L, 8174F, 8512B, 2242B, 2237R, 2236Q, 8837D, 8836C, 2856H, 2444P, 8003F, 1900B, 10234P, 10241B, 9366Y, 10231L, 10245F, 9367B, 9156X, 9155W, 8290H, 3059B, 1628Q, 1627P, 8513C, 8856D, 8883M, 8855C, 9365X, 8857E, 8583R, 8868R, 8302Y, 8301X	N06AA02, N06AA04, N06AA09, N06AA10, N06AA12, N06AA16, N06AB03, N06AB04, N06AB05, N06AB06, N06AB08, N06AB10, N06AB03, N06AF03, N06AF04, N06AG02, N06AX03, N06AX11, N06AX16, N06AX18, N06AX21, N06AX23	–	F32, F33
Anxiety†	3135B, 3134Y, 5355W, 5356X, 5372R, 5371Q, 4144D, 4145E, 3135B, 3134Y, 9432K, 9433L, 10181W, 5357Y, 5358B, 5373T, 5374W, 4150K , 4151L, 4216X, 4522B, 8700X, 8701Y, 8849R	N05BA01, N05BA04, N05BA08, N05BA12, N05BE01	–	F41.1

ATC, Anatomical Therapeutic Chemical Classification; ICT, International Classification of Diseases.

* Non-melanoma skin cancer was excluded in our analysis.

† Pharmaceutical Benefits Scheme (PBS) codes were consistent with the corresponding Anatomical Therapeutic Chemical codes listed in a previous publication based on the 45 and Up Study [8]. We used PBS codes instead of ATC codes for diagnosis detection where each ATC code may include numerous PBS codes. Different PBS codes within one ATC code represent different doses, forms (pill or liquid), intake methods (oral intake or injection), and specific conditions, which helps distinguish the claim purposes for different conditions.

Table S2. Combinations of the hyper-parameters with best performance for random forest*.

Variables	Random Forest
Men	max_depth = 5, mtries = 7, seed = 1, nfolds = 5, ntree = 500
Women	max_depth = 6, mtries = 4, seed = 1, nfolds = 5, ntree = 500
All	max_depth = 6, mtries = 4 seed = 1, nfolds = 5, ntree = 500

* These combinations of the hyper-parameters with best performance would then be separately applied in the machine learning in the final analysis.

Table S3. Area under curve by random forest*.

Variables	Random Forest
Male	0.6597
Female	0.6176
All	0.6592

* We randomly selected 70% to the total population as training data and the remaining 30% as testing data. The testing data were used to evaluate the area under curve for both cross-sectional and longitudinal analysis.

Table S4. Twenty leading predictors by random forest in the longitudinal analysis*.

Ranking	Men		Women		All	
	Predictor	Percentage of variance explained	Predictor	Percentage of variance explained	Predictor	Percentage of variance explained
1	Age	9.0	Smoking	5.4	Age	8.0
2	Relative socioeconomic disadvantage	4.8	Self-rated quality of life	4.7	Self-rated quality of life	5.4
3	Psychological distress	3.8	Self-rated health	4.2	Asthma	4.5
4	Education	3.6	Chicken intake	3.8	Self-rated health	4.4
5	Diabetes	3.5	Red meat intake	3.7	Psychological distress	4.2
6	Smoking	3.5	Psychological distress	3.5	Gender	4.2
7	Self-rated health	3.4	Arthritis	3.3	Red meat intake	3.6
8	Income	3.3	Relative socioeconomic disadvantage	3.2	Smoking	3.6
9	Red meat intake	3.2	Vegetables intake	3.2	Chicken intake	3.4
10	Asthma	2.9	Asthma	3.1	Income	3.3
11	Sleep time	2.9	Alcohol consumption	2.6	Vegetables intake	2.6
12	Sitting time	2.9	Age	2.6	Ancestry	2.4
13	Chicken intake	2.8	Sitting time	2.5	Arthritis	2.4
14	Vegetables intake	2.7	BMI	2.5	BMI	2.4
15	Physical activity	2.6	Income	2.5	Relative socioeconomic disadvantage	2.3
16	Self-rated quality of life	2.5	Working status	2.5	Working status	2.2
17	Fish intake	2.4	Outdoor physical activity	2.4	Alcohol consumption	2.1
18	Rurality	2.3	Milk intake	2.3	Fruit intake	2.1
19	Milk intake	2.2	Fruit intake	2.2	Education	2.0
20	Fruit intake	2.1	Health insurance	2.2	Sitting time	2.0

* Machine learning methods were used to evaluate the importance of predictors in men and women separately and also in the total population.

Table S5. The hazard ratio for incident mental disorders associated with cancer at baseline.

Variable	Events/ Participants	Incidence	HR (95% CI)*	*p*-value
Men	–	–	–	<0.0001
Cancer at baseline	–	–	–	–
No	6016/44,402	13.6	1.00 (1.00–1.00)	–
Yes	840/5077	16.6	1.19 (1.11–1.28)	–
Women	–	–	–	<0.0001
Cancer at baseline	–	–	–	–
No	10,929/53,538	20.4	1.00 (1.00–1.00)	–
Yes	1714/7485	22.9	1.11 (1.05–1.16)	–
All	–	–	–	<0.0001
Cancer at baseline	–	–	–	–
No	16,945/97,940	17.3	1.00 (1.00–1.00)	–
Yes	2554/12,562	20.3	1.15 (1.10–1.20)	–

* Hazard ratios were assessed using Cox regression models adjusted for age, gender, country of birth, income, education, work status, number of parenting children, BMI, psychological distress, hypertension, dyslipidemia, diabetes, asthma, arthritis, hip replacement, and family history of cancer, depression, heart disease, stroke, diabetes, hypertension, Parkinson's disease, and dementia.

Table S6. The hazard ratio for incident cancer associated with mental disorders at baseline.

Variable	Events/Participants	Incidence	HR (95% CI)*	*p* value
Men	–	–	–	0.83
Mental disorders at baseline	–	–	–	–
No	3679/44,402	8.3	1.00 (1.00–1.00)	–
Yes	686/8323	8.2	0.98 (0.90–1.07)	–
Women	–	–	–	–
Mental disorders at baseline	–	–	–	0.39
No	4940/53,538	9.2	1.00 (1.00–1.00)	–
Yes	1759/18,530	9.5	1.03 (0.97–1.10)	–
All	–	–	–	–
Mental disorders at baseline	–	–	–	0.32
No	8619/97,940	8.8	1.00 (1.00–1.00)	–
Yes	2445/26,853	9.1	1.03 (0.98–1.08)	–

* Hazard ratios were assessed using Cox regression models adjusted for age, gender, country of birth, income, education, work status, number of parenting children, BMI, psychological distress, hypertension, dyslipidemia, diabetes, asthma, arthritis, hip replacement, and family history of cancer, depression, heart disease, stroke, diabetes, hypertension, Parkinson's disease, and dementia.

References

1. Australian Institute of Health and Welfare. The Active Australia Survey: a guide and manual for implementation analysis and reporting, Canberra, Australia, 2003.
2. Australian Bureau of Statistics. 2039.0–Information paper: an introduction to socio-economic indexes for areas (SEIFA), 2006; Canberra, Australia, 2008.
3. Australian Population and Migration Research Centre. *Accessibility/Remoteness Index of Australia*; Australian Population and Migration Research Centre: Adelaide, Australia, 2012.
4. Kessler, R.C.; Andrews, G.; Colpe, L.J.; Hiripi, E.; Mroczek, D.K.; Normand, S.-L.T.; Walters, E.E.; Zaslavsky, A.M. Short screening scales to monitor population prevalences and trends in non-specific psychological distress. *Psychol. Med.* **2002**, *32*, 959–976, doi:10.1017/s0033291702006074.
5. Goodger, B.; Byles, J.; Higganbotham, N.; Mishra, G. Assessment of a short scale to measure social support among older people. *Aust. New Zealand J. Public Heal.* **1999**, *23*, 260–265, doi:10.1111/j.1467-842x.1999.tb01253.x.
6. Breiman, L. Random forests. *Machine Learning* **2001**, *45*, 5–32.
7. Kuhn M; Johnson K. *Applied predictive modeling*; Springer: New York, NY, USA, 2013.
8. Lujic, S.; Simpson, J.M.; Zwar, N.; Hosseinzadeh, H.; Jorm, L. Multimorbidity in Australia: Comparing estimates derived using administrative data sources and survey data. *PLoS One* **2017**, *12*, e0183817, doi:10.1371/journal.pone.0183817.

© 2020 by the authors. Licensee MDPI, Basel, Switzerland. This article is an open access article distributed under the terms and conditions of the Creative Commons Attribution (CC BY) license (http://creativecommons.org/licenses/by/4.0/).
